# Cerebral and somatic oxygen saturation in pediatric cardiac patients with delayed sternal closure

**DOI:** 10.1186/2197-425X-3-S1-A950

**Published:** 2015-10-01

**Authors:** L Kovacikova, A Bicianova, P Skrak, M Zahorec

**Affiliations:** National Institute of Cardiovascular Diseases, Pediatric Cardiac Intensive Care Unit, Bratislava, Slovakia

## Introduction

Near-infrared spectroscopy offers non-invasive online monitoring of tissue oxygenation in a wide range of clinical scenarios.

## Objectives

The purpose of this study was to assess near-infrared spectroscopy-derived cerebral and somatic oxygen saturations (rSO_2_b and rSO_2_s) in children after cardiac surgery with delayed sternal closure (DSC). We hypothesized that rSO_2_b and rSO_2_s correlate with other indicators of hemodynamic compromise after DSC.

## Methods

We studied 43 postoperative children (median age, 8 days; range, 1-148 days) with DSC. The most common cardiac diagnosis was hypoplastic left heart syndrome (30%). rSO_2_b and rSO_2_s and other hemodynamic and metabolic parameters were analyzed on the day of surgery, 3 days after surgery (POD1-3), and prior to and 24 hours after DSC.

## Results

rSO_2_b increased at 12 hours (p = 0.0039), and rSO_2_s did not change compared to data immediately after surgery. Deficit base decreased (p = 0.016) and standard bicarbonates increased (p = 0.016) on POD1, lactate decreased (p = 0.0001) and diuresis increased (p = 0.013) on POD2, heart rate decreased (p = 0.035) on POD3. There was no change in systemic arterial blood pressure, left atrial pressure (LAP) and central venous pressure (CVP), arteriovenous oxygen saturation difference (a-vO2 dif.), pH, paO_2_, and paCO_2_. Vasoactive inotropic score was lower on POD2 and 3 compared to the day of surgery (p = 0.041 and p = 0.048). DSC resulted in an increase in LAP, CVP, a-vO2 dif., and base deficit (p < 0.05). Increase in LAP lasted 24 hours, other changes were present during 18 hours. rSO_2_b and rSO_2_s were lower at 1 - 6 hour and during 24 hours, respectively (p < 0.05). There was no change in lactate levels. pH and standard bicarbonate were lower and vasoactive inotropic score was higher compared to preclosure values (p < 0.05). rSO_2_b and rSO_2_s values did not correlate with lactate and a-vO2 dif. values.

## Conclusions

In pediatric cardiac patients with left open chest rSO_2_b values increase on the day of surgery and rSO_2_s do not change. DSC is associated with a decrease in rSO_2_b and rSO_2_s that persists for 6 and 24 hours, respectively. rSO_2_b and rSO_2_s values do not correlate with other metabolic indicators of hemodynamic status.
